# Dr. John W. Fawdry

**Published:** 1916-01

**Authors:** 


**Dr. John W. Fawdry,** N. Y. Homoeopathic 1892, died at his home in Corfu, November 10, aged 50.

